# Acroangiodermatitis following sclerotherapy: A rare complication

**DOI:** 10.1016/j.jvscit.2025.101920

**Published:** 2025-07-10

**Authors:** Paula Dias, Juliana Sousa, Maria José Julião, Ricardo Vale Pereira, Manuel Fonseca

**Affiliations:** a Angiologia e Cirurgia Vascular, ULS Coimbra, Coimbra, Portugal; b Anatomia Patológica, ULS Coimbra, Coimbra, Portugal

**Keywords:** Sclerotherapy, Aged, Vascular malformations, Doppler ultrasonography, Histopathology

## Abstract

Sclerotherapy is a widely used treatment for varicose veins; although numerous complications are documented, delayed complications are uncommon. This report describes an 82-year-old woman who developed a firm, nonpulsatile leg mass 3.5 years after ultrasound-guided sclerotherapy. Doppler ultrasound examination suggested a varicose collateral aneurysm, but atypical features and the risk of malignancy prompted surgical excision. Histopathology revealed a benign reactive vascular lesion, likely related to prior endothelial injury and thrombosis. This case highlights the importance of considering previous interventions when evaluating new vascular lesions and demonstrates that, although imaging is useful, histopathological evaluation is essential for accurate diagnosis and management.

Sclerotherapy is a minimally invasive chemical treatment used to manage varicose veins and venous malformations. It involves the injection of a sclerosing agent such as polidocanol or sodium tetradecyl sulphate into the affected vein, which induces endothelial damage, leading to inflammation, protein denaturation, cellular dehydration, and luminal obstruction. These processes result in sclerosis of the targeted vein, hence the term sclerotherapy. This method is effective for treating both main and smaller branch veins and is particularly beneficial for residual veins after surgery, offering high clinical success rates.^1^

Although generally considered safe, sclerotherapy can lead to complications. Mild side effects such as transient hyperpigmentation, telangiectatic matting, superficial venous thrombosis and neurological events, such as visual disturbance, dysesthesia, headache, and migraine, are frequently most frequently reported and typically self-limited. More serious, though uncommon, complications include deep vein thrombosis, anaphylactic shock, transient ischemic attack, and stroke. Rarely, issues may arise from the treatment spreading into deeper veins or owing to accidental injection into an artery, which can result in more severe outcomes like tissue necrosis or limb damage.^2^, ^3^, ^4^

Most adverse effects occur early and are manageable with conservative care, and delayed or long-term complications are uncommon.^5^ These rare cases underscore the importance of careful technique, proper patient selection, and close follow-up.^6^ This case report adds to the understanding of sclerotherapy's potential risks by presenting an elderly patient who developed a complex vascular lesion approximately 3 years after undergoing sclerotherapy. Written informed consent was obtained from the patient for the publication of this case report and accompanying images.

## Case report

An 82-year-old woman with a medical history of aortic stenosis, omarthrosis, urinary incontinence, and prior superficial venous thrombosis presented to the emergency department with a new complaint. She had been treated for symptoms related to superficial venous reflux after superficial venous thrombosis 3.5 years prior. This treatment had been done with polidocanol as ultrasound-guided foam (physician-compounded) sclerotherapy in the right anterolateral lower leg. No immediate complications were reported after the procedure.

**Fig 1:**
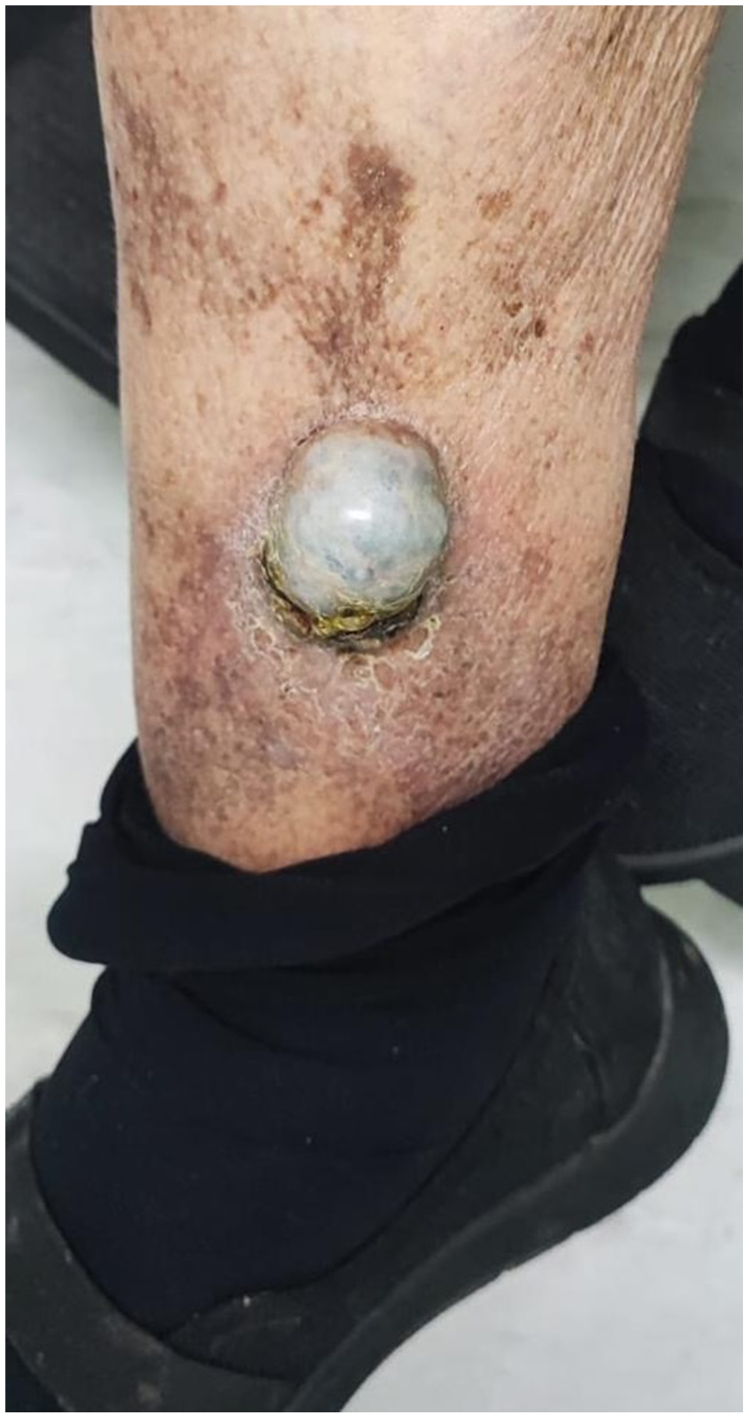
Presentation of sac-like mass on the distal one-third of the right leg in the emergency department.

The patient presented with a sac-like mass on her right leg (Fig 1). She reported gradual growth over several months after sclerotherapy without significant pain or systemic symptoms. On physical examination, a protruding, firm, nonpulsatile mass measuring approximately 3 to 4 cm was observed on the distal one-third of the right leg. Based on clinical findings and Doppler ultrasound evaluation, a large varicose collateral aneurysm was suspected.

**Fig 2:**
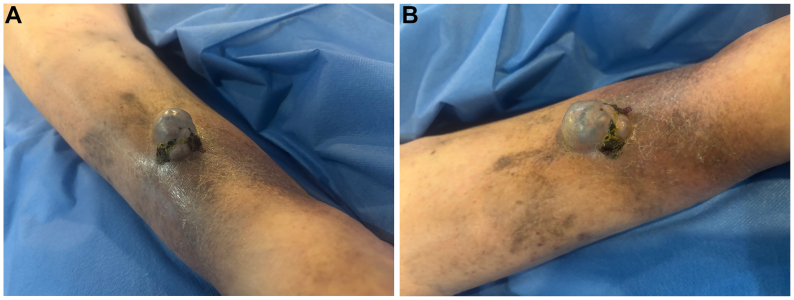
**(A** and **B)** Preoperative evaluation of the protruding, firm, nonpulsatile mass with hemorrhagic crusting.

Preoperative evaluation revealed a dermal swelling measuring 3 × 4 cm with a predominantly firm consistency and partial compressibility under pressure, with subsequent refilling, which suggested a venous malformation (Fig 2). Additionally, hemorrhagic crusting was noted along the lesion's borders.

**Fig 3:**
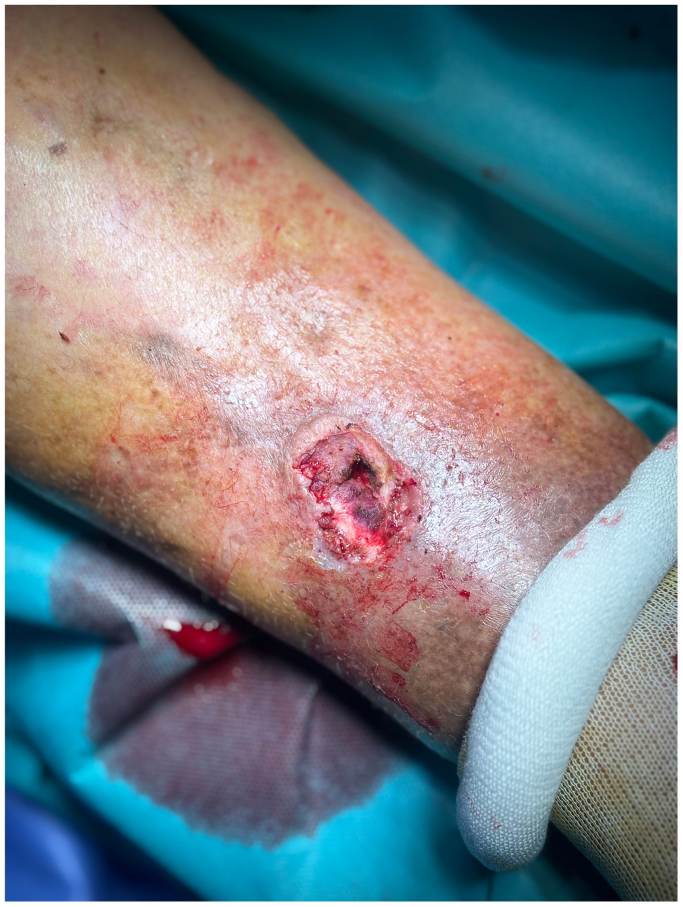
Immediate postoperative wound after lesion excision.

Given the atypical findings and the potential risk of malignancy or underlying pathology, surgical excision was performed. The lesion was excised successfully under local anesthesia approximately 3 months after the initial presentation. Hemostasis was achieved by ligating hemorrhagic points at the lesion's base using 6-0 monofilament polypropylene sutures (Fig 3). The excised tissue was sent for histopathological analysis to rule out malignancy. Postoperatively, the wound was dressed, and no immediate or 24-hour complications were observed.

**Fig 4:**
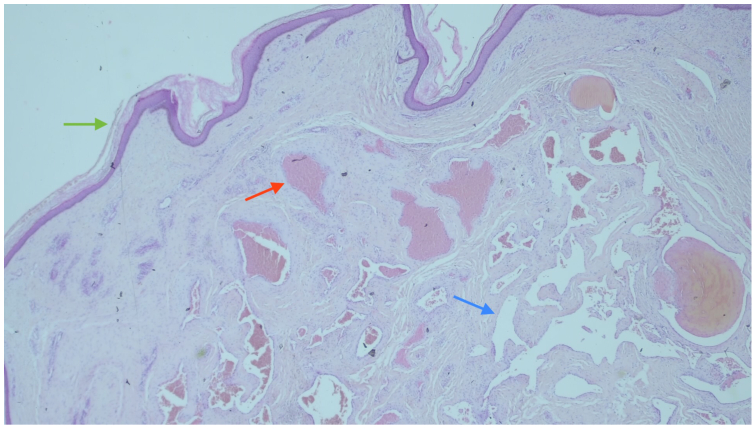
Microscopic image of the excised tissue stained with hematoxylin and eosin at an original magnification of ×40. The *green arrow* indicates the thickened epidermis and lobular capillary growth pattern in the superficial dermis. The *red arrow* highlights dilated, irregular blood vessels lined by bland endothelial cells without atypia, consistent with a benign reactive proliferation. The *blue arrow* shows an area of recanalized vessel or papillary endothelial hyperplasia, a common reactive change after thrombosis.

**Fig 5:**
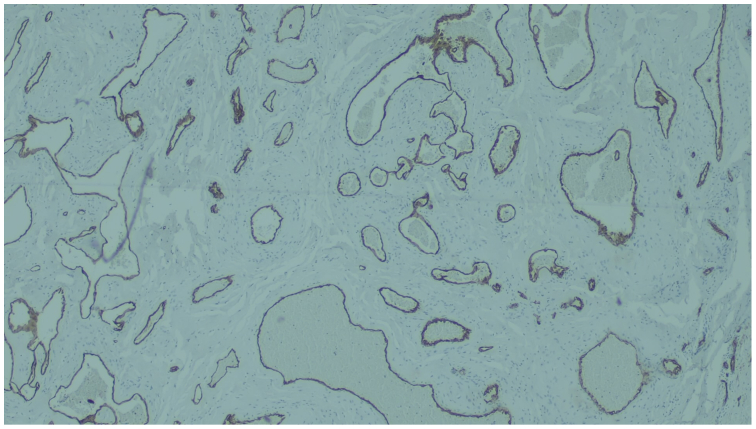
Microscopic image of the excised tissue stained with immunohistochemical CD34 antibody at an original magnification of ×40.

Histopathological analysis revealed a benign reactive vascular lesion. Microscopically, there was a proliferation of dilated, tortuous blood vessels lined by regular, nonatypical endothelial cells, with no signs of malignancy. Immunohistochemical staining demonstrated positive expression of ERG and CD34, confirming the vascular nature of the endothelial cells. The overlying skin and the superficial dermis resembled an acroangiodermatitis, a condition linked to chronic venous stasis or previous vascular procedures such as sclerotherapy. These findings supported a diagnosis of a reactive vascular proliferation (Figs 4 and 5).

## Discussion

This case highlights a rare long-term complication of sclerotherapy, specifically the development of a benign reactive vascular lesion masquerading as a varicose collateral aneurysm. The findings emphasize that, even years after an otherwise successful procedure, patients can present with unexpected vascular changes that require a thorough clinical assessment.

Although sclerotherapy is generally considered safe and effective, delayed complications can occur.^1^ The lesion's firm consistency, partial compressibility, and hemorrhagic crusting suggested a venous malformation rather than a simple varicose aneurysm. This atypical presentation merited histopathological evaluation to rule out malignancy or other underlying pathologies.

Histopathological examination ultimately revealed a benign reactive vascular lesion, resembling a cavernous hemangioma. However, unlike cavernous hemangiomas, which are typically congenital and characterized by large, dilated, blood-filled spaces, this lesion exhibited reactive changes such as thrombosis and fibrosis. These findings suggest that sclerotherapy may lead to local endothelial damage, thrombosis, and subsequent abnormal vascular remodeling, potentially mimicking or triggering vascular anomalies.^7^^,^^8^

Other contributing factors must also be considered. The lesion occurred on the anterior tibial region, a sun-exposed area, and although the patient did not have ulceration, she had a history of superficial venous thrombosis and chronic venous insufficiency. These conditions can lead to altered tissue oxygenation, venous hypertension, and local inflammatory responses, all of which may contribute to aberrant vascular remodeling. Ultraviolet radiation, although more commonly linked to epithelial neoplasms, may play a supplementary role in promoting endothelial changes, particularly in the setting of compromised vascular dynamics.^9^^,^^10^

Importantly, this case illustrates that Doppler ultrasound examination, although useful as an initial evaluation tool, may not be sufficient for a definitive diagnosis, particularly when lesions exhibit unusual morphology or behavior. In such cases, histopathological analysis remains the gold standard for accurate diagnosis and to exclude malignancy. The benign nature of the lesion in this patient reassures clinicians that, despite its atypical appearance and growth pattern, malignancy was not a concern.

Finally, this case underscores the importance of thoroughly evaluating a patient's medical history when assessing atypical vascular lesions. Identifying prior interventions—such as sclerotherapy—can provide critical context for interpreting the lesion's nature and guiding further diagnostic steps. Recognizing a potential association between past procedures and current vascular changes can help clinicians to distinguish between benign reactive processes and more concerning pathologies, ensuring that appropriate diagnostic and therapeutic decisions are made.

## Conclusions

This case emphasizes the importance of considering a patient's past medical history and previous intervention when evaluating soft tissue lesions. Although imaging can guide initial assessment, histopathological analysis remains essential for definitive diagnosis. Although rare, delayed benign vascular changes can occur and should be managed through careful clinical evaluation and appropriate surgical and pathological investigations.

## Declaration of generative Ai and Ai-assisted technologies in the writing process

During the preparation of this work, the authors used ChatGPT to improve readability. After using this tool/service, the authors reviewed and edited the content as needed and take full responsibility for the content of the publication.

## Funding

None.

## Disclosures

None.
